# The effects of a two-week neuromuscular intervention on biopsychosocial variables in people with patellofemoral pain: an observational study

**DOI:** 10.3389/fspor.2023.1087061

**Published:** 2023-05-15

**Authors:** Simon David Lack, Clare Bartholomew, Thomas North, Stuart Charles Miller, Bradley Stephen Neal

**Affiliations:** ^1^ Sports and Exercise Medicine, Queen Mary University London, Mile End Hospital, London, United Kingdom; ^2^ Pure Sports Medicine, Point West Building, London, United Kingdom; ^3^ School of Sport, Rehabilitation and Exercise Science, University of Essex, Colchester, Essex, United Kingdom

**Keywords:** patellofemoral, kinematic, biopsychosocial, electromyography, rehabilitation

## Abstract

**Introduction:**

Patellofemoral pain (PFP) is common and predominately affects active populations. Altered biomechanics and psychosocial variables have been reported in people with PFP, but the effects of neuromuscular exercise on these variables is unknown. We aimed to investigate changes in biopsychosocial measures following a two-week neuromuscular intervention in people with PFP.

**Materials and Methods:**

We measured pain (visual analogue scale), function (Kujala), activity level (Tegner), psychological well-being (Orebro), lower-limb isometric strength (handheld dynamometry), three-dimensional (3D) lower limb kinematics, and surface electromyography (sEMG), in people with PFP. 3D lower-limb kinematics and sEMG were synchronously sampled during step-up, step-down, and overground running. All measures were repeated after participants had completed a two-week neuromuscular intervention consisting of three exercises completed once per day, five days per week.

**Results:**

18 participants completed pre/post testing (60% females, mean age 30.6 years ±7.0, height 173.4cm ±10.4, mass 70.2kg ±12.4, symptom duration 39.0 months ±58.8), with three of 21 participants lost to follow-up. Across all clinical measures (muscle onsets, muscle activation and kinematics), the 95% bootstrapped confidence intervals (CI) of the mean difference contained the null hypothesis following the two-week neuromuscular intervention, indicating no significant differences.

**Conclusion:**

A two-week neuromuscular intervention did not change biomechanical or psychosocial measures in people with PFP. Interventions with a longer duration or greater load magnitude are required to fully evaluate the biopsychosocial mechanisms of effect for exercise in people with PFP.

## 1. Introduction

Patellofemoral pain (PFP) is characterised by pain at, around, or behind the patella during activities that load the patellofemoral joint, such as stair ambulation and running (1). PFP is common; accounting for up to 25% of all knee injury presentations to sports medicine clinics (2). The aetiology of PFP is unknown (3), with limited evidence indicating that a variety of local structures may contribute to nociception (4). An absence of greater treatment success (5) may be due to the underlying factors that contribute to the development of PFP not being adequately addressed, or that treatment targets differ between people. Evaluating the biopsychosocial mechanisms of effect for specific interventions provides insight into the ability to change specific variables, or the variability of response, within individuals.

Altered lower-limb biomechanics that affect load distribution across the patellofemoral joint have been purported to result in supra-physiological overload (6); contributing to the nociceptive cascade that may result in PFP symptoms. Distal (foot), local (knee), and proximal (hip and trunk) factors contribute to these altered lower limb biomechanics and have been observed to differ in people with PFP (7). Increased hip adduction and internal rotation during functional tasks, and weaknesses of the quadriceps, hip abductor, and hip external rotator muscles, are commonly reported in people with PFP (8, 9). Level one evidence also reports delayed vastus medialis oblique onset relative to vastus lateralis, and shorter durations of gluteal neuromuscular activity, when people with PFP climb stairs and run (10, 11). Rehabilitation interventions directed proximal to the patellofemoral joint have established efficacy in people with PFP but the mechanism of effect for these interventions is unknown (12, 13). Neurophysiological changes within skeletal muscle in response to resistance exercise have been reported to occur after short training periods (i.e., ≤2-weeks) in people without knee pain (14, 15), but the time period or effects have not been investigated in people with PFP. Evaluating the biomechanical effects of resistance training in people with PFP is necessary to guide the clinical prescription of exercise interventions.

Psychosocial features, including heightened levels of anxiety, depression, catastrophising, and fear avoidance, have all been reported in people with PFP (16). Due to a limited number of published studies, the 2018 consensus statement of clinical-academic leaders with a special interest in people with PFP, highlighted the need for future work to investigate the effects of interventions on psychosocial impairments (17). Long symptom duration and recalcitrance to treatment are often observed in studies reporting long-term follow up (18, 19), characteristics that are associated with greater psychosocial burden. Identification of interventions that have positive effects on psychosocial variables is clearly a priority to ensure that all factors contributing to symptoms are being adequately addressed.

With altered lower limb kinematics and neuromuscular activation of the gluteal region evident in people with PFP, a plausible mechanism of effect for proximal rehabilitation exercise may be through changes in these biomechanical domains, and may start to occur after as little as two-weeks of exercise. Similarly, with anxiety, depression, catastrophising and fear avoidance elevated in people with PFP (16), and resistance exercises suggested to be of positive effect (20), improvements in pain and function may be attributable to changes in the psychosocial domain. The aim of this study was therefore to explore if a two-week neuromuscular intervention, as defined by the American College of Sports medicine (21), led to changes in biomechanical and/or psychosocial variables in people with PFP. The null hypothesis being a two-week neuromuscular intervention will not result in biomechanical and/or psychosocial change in people with PFP.

## 2. Materials and methods

Ethical approval was obtained through the Queen Mary Ethics of Research Committee (QMREC2014/24/103).

### 2.1. Sample size

An *a priori* sample size was calculated using G*Power programme (https://g-power.apponic.com/). Using previously published data (22) comparing activation duration of the gluteus medius during stair ascent in people with and without PFP (control mean 758.8 ms ±115.7, PFP mean 608.1 ms ±206.4), a sample size of 21 was required to achieve *α* = 0.05 and *β* = 0.8, with an anticipated effect size of 0.9.

### 2.2. Participants

Participants were recruited through two private sports medicine clinics (Pure Sports Medicine and Nuffield Health, London, UK) and were assessed for eligibility by a physiotherapist with >10 years clinical experience (SL). Participants were eligible if they were (i) aged 18–40 years, (ii) reported insidious onset of symptoms present for ≥6 weeks, and (iii) had symptoms that were aggravated by ≥2 of the following—prolonged sitting or kneeling, squatting, running, or stair ascent/descent. Participants were ineligible if they demonstrated (i) concomitant injury/pain from the hip, lumbar spine or other knee structures, (ii) previous knee surgery, (iii) patellofemoral instability, (iv) intra-articular pathology or joint effusion, (v) physiotherapy or foot orthoses use in the preceding year. Written informed consent was obtained from all eligible participants prior to data collection.

### 2.3. Procedure overview

Eligible participants attended the human performance laboratory at Queen Mary University London, for the collection of demographics and measures of symptoms, function, and strength. Functional tasks, including step up, step down, and running, whilst having lower limb biomechanical data sampled, were performed. Participants then completed a two-week neuromuscular intervention, after which the test procedure was repeated.

### 2.4. Symptoms, function, and strength

#### 2.4.1. Pain

Horizontal 100 mm visual analogue scales (VAS), anchored by “no pain” (0 mm) and worst pain imaginable' (100 mm), were used to measure usual and worst pain over the previous week, with a change in score of 15 mm considered a minimum clinically significant difference (23).

#### 2.4.2. Function

The Anterior Knee Pain Scale (Kujala), a 13-item patient-reported measure related to symptoms and varying levels of current knee function, such as ability to perform stairs, walk, run, jump and sit for prolonged periods, was used to measure function (15). Each item is weighted and a total score between 0 and 100 is calculated, with higher scores representing greater levels of function, and a minimum clinically significant difference of 10 points (24).

#### 2.4.3. Activity level

Tegner score, a self-reported measure of activity, was used to quantify activity levels of participants (25). Scores range from Level 0 (sick leave or disability pension) to Level 10 (national elite competitive sports player).

#### 2.4.4. Psychosocial well-being

Psychosocial well-being was assessed using the Orebro Musculoskeletal Pain Screening Questionnaire (26). It consists of 25 self-reported items and is helpful to evaluate pain, function and time off work due to sickness caused by musculoskeletal conditions. The maximum score is 210 points; a score of <105 points indicates a low disability, that between 105 and 130 points indicates a moderate disability and that >130 points indicates a high disability (26).

#### 2.4.5. Lower-limb isometric strength

Measures of isometric hip extension and external rotation strength were recorded using a handheld dynamometer (Lafayette Manual Muscle Tester, Lafayette, USA). Using previously described test positions (27), hip extension was assessed with participants in prone and the knee flexed to 90°. A webbing strap was wrapped around the couch, and the dynamometer placed on the distal third of the posterior thigh, under the strap. Participants were instructed to “push their heel to the sky” as firmly as possible. Hip external rotation was assessed with participants in sitting, with the hip and knee flexed to 90°. The webbing strap was placed around the base of the couch, with the dynamometer placed on the distal third of the medial tibia, maintaining the hip in a neutral rotation position. The participant was instructed to “pull the ankle towards the opposite ankle” as firmly as possible. The addition of web strapping to stabilise the dynamometer was incorporated into the tests to minimise the risk of the examiner being overpowered (28, 29). Participants were given the opportunity to practice the test manoeuvre until the examiner was happy that they could perform the test with the correct technique. For both test positions, participants were encouraged to push maximally into the handheld dynamometer for five seconds over three repetitions, with 15 s rest between each. The highest of the three scores was recorded to reflect a maximum voluntary contraction (MVC) for all tests.

### 2.5. Biomechanical data collection

#### 2.5.1. Surface electromyography

Wireless surface EMG (sEMG) electrodes (Delsys, Trigno) were attached as per the surface electromyography for the non-invasive assessment of muscles (SENIAM) guidelines (30). A single bi-polar rectangular electrode (27 × 37 × 13 mm) with an intra-electrode distance of 10 mm was placed over each muscle. Prior to electrode placement the skin of the targeted area was shaved, cleaned with an alcohol wipe, and allowed to air dry. Surface electrodes were placed on the symptomatic side only. If both knees were affected the most symptomatic side was used. Electrodes were placed over the anatomical region of the tensor fascia lata (TFL), gluteus medius (GMed), upper fibres of gluteus maximus (UGmax), lower fibres of gluteus maximus (LGmax), vastus lateralis (VL), and vastus medialis oblique (VMO). TFL electrode placement was on the line between the anterior superior iliac spine and the lateral femoral condyle, in the proximal 1/6th. The GMed electrode was placed 50% along the line from the crista iliaca to the greater trochanter. A perpendicular line was drawn at the midpoint of a line joining the sacral vertebrae and the greater trochanter. The UGMax electrode was placed 2 cm superiorly and laterally along this line, whilst the LGMax electrode was placed 2 cm inferiorly and medially. The VL electrode was placed 2/3 on the line from the anterior superior iliac spine to the lateral aspect of the patella, and the VMO electrode was placed 80% on the line between the anteromedial tibiofemoral joint line and the anterior superior iliac spine.

#### 2.5.2. 3D lower-limb kinematics

Participants were initially provided with neutral running shoes in their required size (Asics Nimbus, Asics, Cheshire, UK), to minimise potential effects of footwear variation on lower limb kinematics (22). Kinematic data were collected during all tasks using a four-camera, infrared motion analysis system using Odin software (CX-1, Codamotion, Charnwood Dynamics Limited, Leicestershire, UK) sampling at 200 Hz. 24 infrared markers, consisting of eight individual markers and four rigid clusters of four markers, were placed on standard pelvic and lower limb anatomical landmarks using the CAST protocol (Cappello et al., 1997) by two investigators (SL or BN). Unpublished laboratory data for two authors (SL & BN) have previously identified moderate to excellent intra-rater reliability with respect to positioning of kinematic markers in three-dimensional space (ICC 0.62–0.93).

Individual markers were applied using double-sided adhesive tape and secured with transparent surgical tape, and placed bilaterally on the anterior superior iliac spine, posterior superior iliac spine, lateral calcaneal process, and the head of the fifth metatarsal. Rigid clusters were applied bilaterally on the mid-point of each thigh and shank segment using adjustable elastic straps and were secured with cohesive self-adherent bandage. Virtual markers were also identified on the femoral epicondyles and the ankle malleoli, to allow for the calculation of relevant joint centres during an upright standing calibration trial that did not differ between male and female participants. The knee joint centre was estimated as the mid-point between the femoral epicondyle markers and the hip joint centre was estimated as a projection within the pelvis frame using previously described methods (31).

#### 2.5.3. Step-up and step-down task

**Figure 1:**
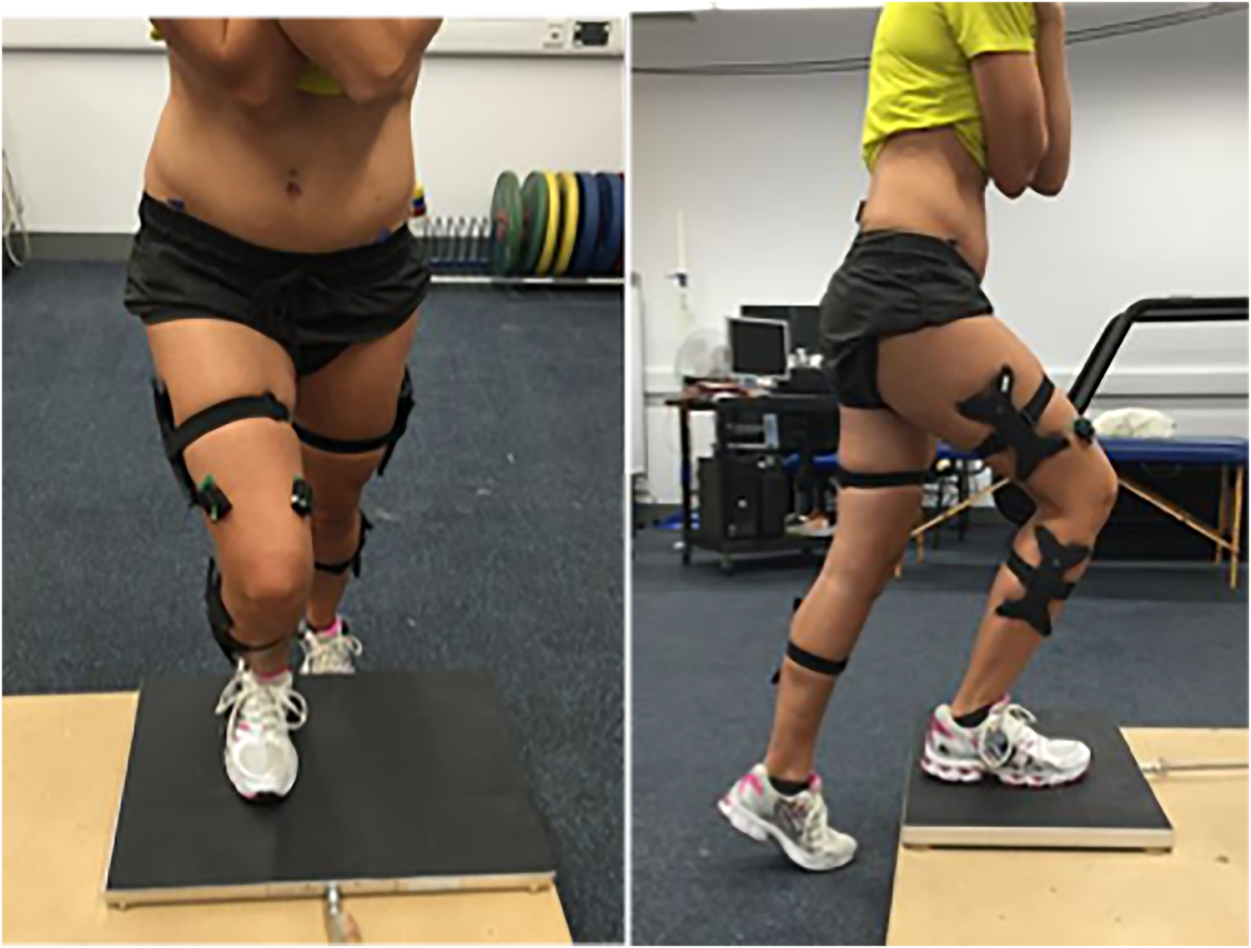
Participant performing the step up task onto the force plate in the laboratory.

A similar protocol to one described previously (32) was used. A Kistler force plate (Type 9281CA, Kistler Corporation, Switzerland) sampling at 1,000 Hz, was mounted on a wooden step (combined height 22 cm; Figure 1). Participants stepped onto the force plate leading with the affected leg, lifting the unaffected leg up to the step, and then stepping back down leading with the affected leg. A total of five continuous repetitions were completed at a self-selected speed during which synchronised sEMG, kinematic, and kinetic were collected. The participants completed the step-down task, starting from the top of the step and force plate, leading with the unaffected leg, to the floor, then stepping backward up onto the step with the unaffected leg. A further five continuous repetitions were completed whilst synchronised EMG, kinematic, and kinetic data were collected.

#### 2.5.4. Overground running

**Figure 2:**
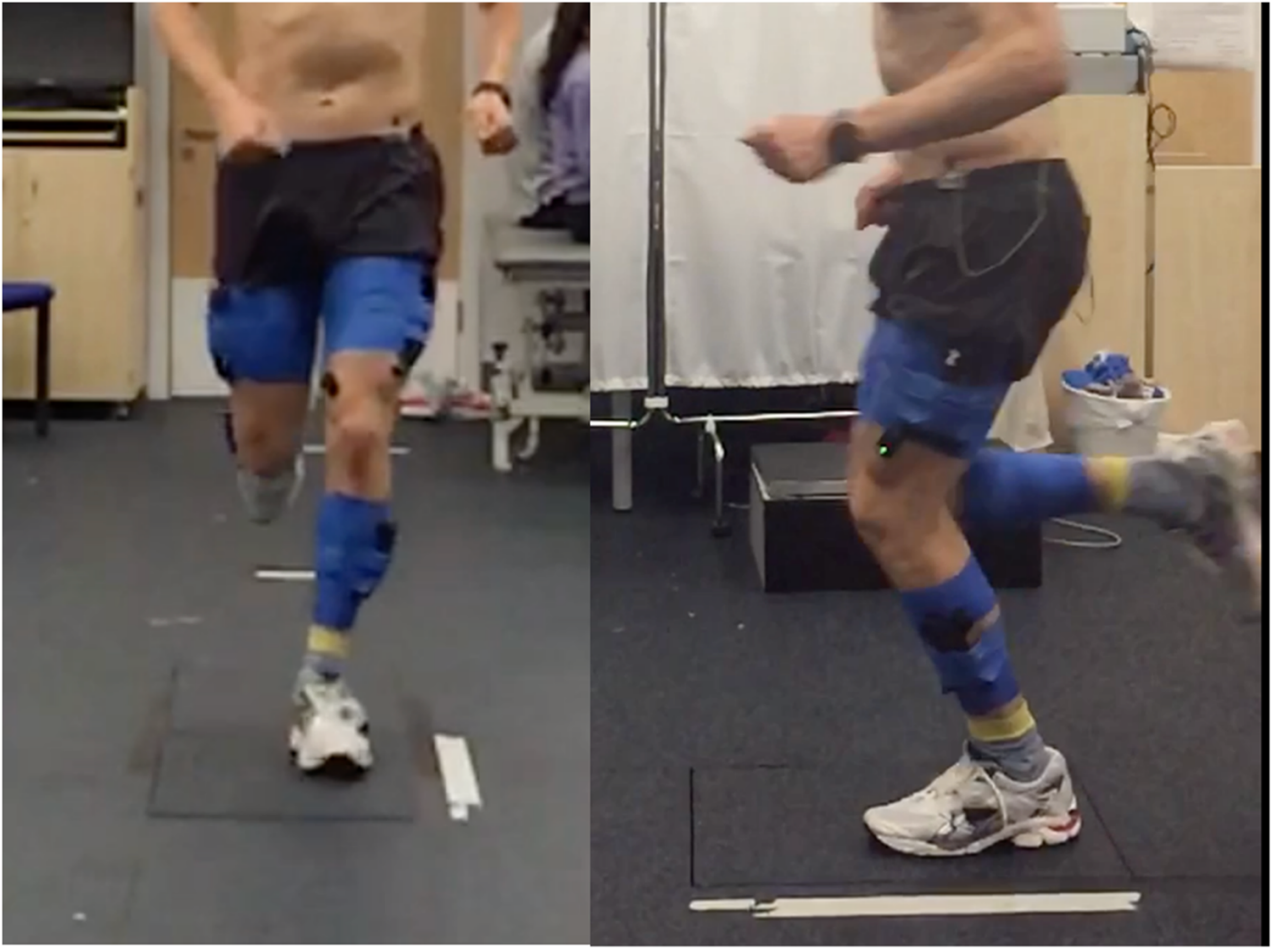
Still image from a running trial, with the participant landing on the ground embedded force plate.

Participants were instructed to run in a straight line for ten meters at a self-selected speed, landing the foot of their symptomatic limb on a ground-embedded force plate sampling at 1,000 Hz (Type 9281CA, Kistler Corporation, Switzerland). The ground-embedded force plate was 5 m from the trial start-point, with participants typically making contact with their fifth step. Several practice trials were permitted to allow for familiarisation and to ensure adequate force plate contact during a participant's natural running gait. This process was repeated until five successful trials were obtained, with a successful trial defined as an appropriate landing of the correct foot directly onto the force plate without obvious adjustment of running gait (Figure 2). 60 s rest between trials was provided. Each trial was initiated by verbal countdown by a member of the research team.

**Figure 4:**
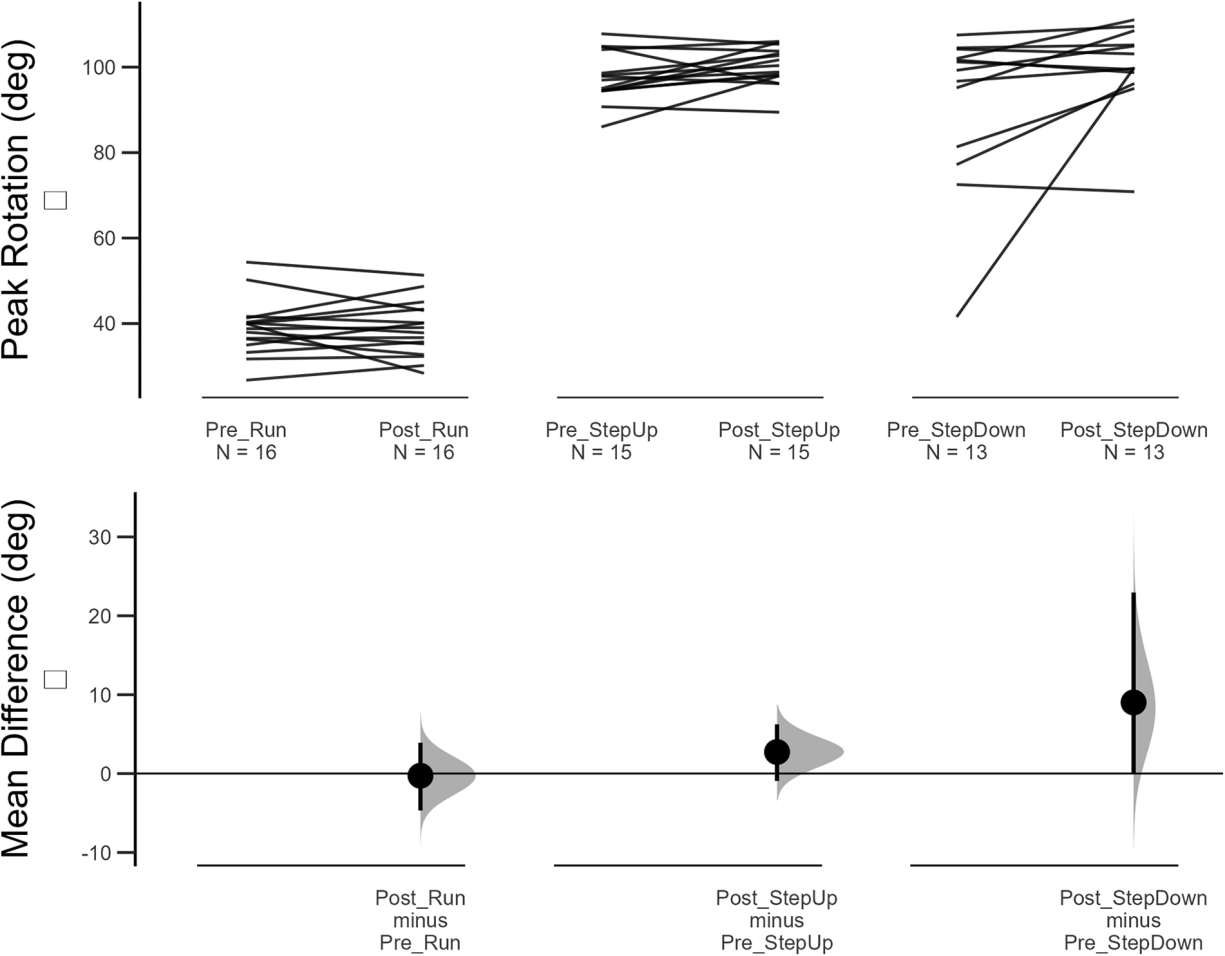
Changes in peak knee flexion pre-post 2 week intervention for running, step up and step down tasks. Top figure shows induvial changes and lower figure showing mean difference and 95% confidence intervals of bootstrapping analysis.

### 2.6. Two-week neuromuscular intervention

**Figure 3:**
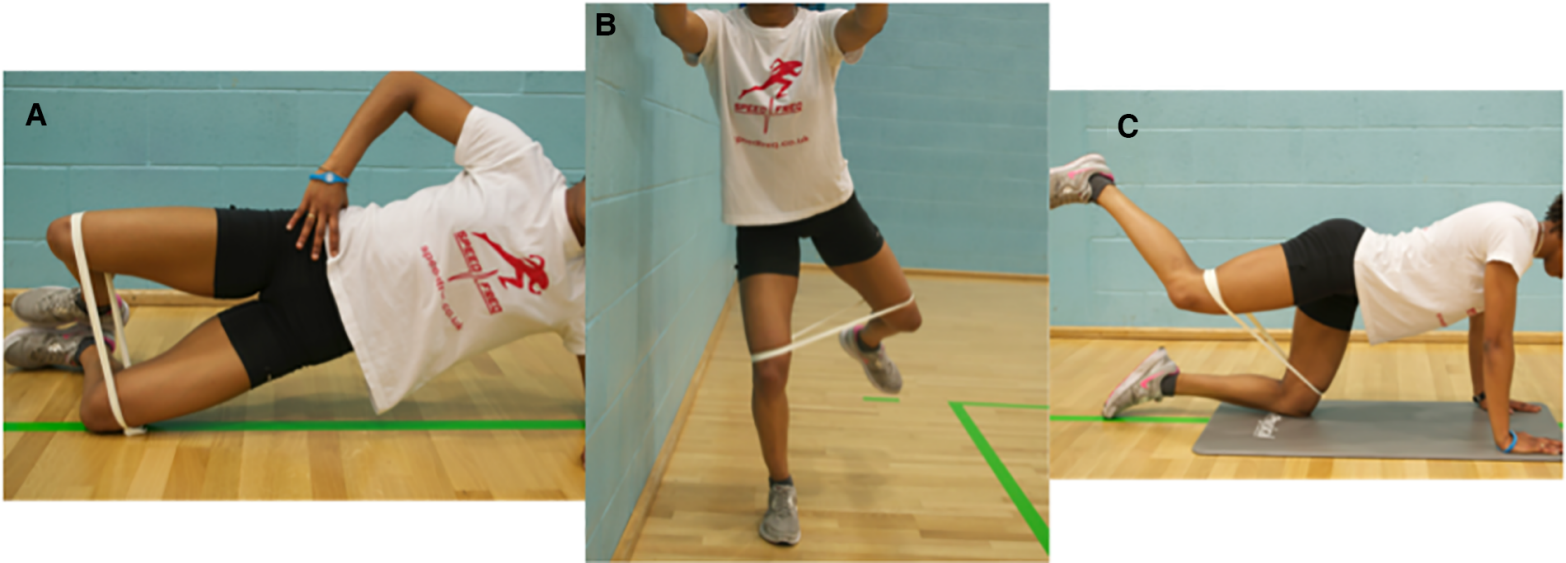
The three exercises performed by the participants. (**A**) The side bridge clam. (**B**) The standing fire hydrant. (**C**) The 4-point fire hydrant.

Participants were prescribed a two-week neuromuscular intervention adhering to the prescription principles defined by the American College of Sports Medicine (21); initially taught by a physiotherapist (SL) with >10 years clinical experience. Participants performed three exercises; the side bridge clam (4 s concentric and 4 s eccentric), four-point fire hydrant (4 s concentric and 4 s eccentric), and the standing fire hydrant (1 s concentric and 1 s eccentric) as shown in Figure 3. Each exercise was repeated five out of every seven days for two consecutive weeks. The time each exercise was performed increased from 10 s to 30 s over the two-week period. Progression was based on the participants self-perceived ability to perform the exercise with good technique (as guided by the therapist) whilst working at an intensity of 40%–50% of their perceived maximum effort within the target muscle (“the gluteal region”). A loop of resistance band with no stretch (“Light” Green, RockbandRx, RockTape®), was tied just above their knees whilst the participant stood with their legs together. Once a participant could perform each exercise for three sets of 30 s holds without the band, they used the resistance band positioned just above the knee, for all exercises. With the band in place, they again started with three sets of 10 s, building to 30 s to ensure a perceived intensity of work was maintained between 40%–50%. To provide further guidance and monitor adherence, participants were provided with an exercise sheet including images of the exercises, a prescription parameter table, and a training diary, but had no further contact with a physiotherapist between baseline and follow up.

Following completion of the two-week neuromuscular intervention participants were invited back to the human performance laboratory to repeat the test protocol. All participants were required to complete the follow-up testing session within a maximum one-week period following the completion of the intervention. During the follow-up session, participants completed isometric strength measures (hip extension and external rotation), sEMG, and kinetic/kinematic data collection during the same step-up, step-down and run tasks as described above.

### 2.7. Outcome measures

The outcomes measured before and after the two-week intervention period were: Orebro and Kujala scores, hip external rotation and hip extension isometric strength, and the root mean square and onset timing (ms) for the four sEMG electrodes (TFL, GMed, UGmax and LGmax), and peak hip adduction, knee internal rotation and flexion during step-up, step-down and running tasks.

### 2.8. Data processing

Raw EMG was initially filtered using a 50 Hz notch ideal filter before being passed through a 20–500 Hz bandpass ideal filter. Kinematic marker data were low pass filtered at 20 Hz. Initial contact, for each step or run, was defined as a force plate reading exceeding 10 N which was not filtered. Analysis occurred within a time window of 0.5 s pre and post initial contact. Muscle activation onset was determined when a threshold was exceeded. For each participant the threshold equalled the minimum of the means of all trials plus 30% of the range (mean of the minima minus the mean of the maxima) (32). Negative values indicated activation occurred before initial contact, with positive values indicating activation occurred after initial contact. The root mean square (RMS) of the signal during this time window was calculated between the muscle onset and its offset (the time when activity fell below the individual's threshold >30 ms). For each participant the timing for muscle activation onset relative to the initial contact was measured for each step or run. Data collected from five trials were averaged and this result was used for data analysis.

### 2.9. Data analysis

Processing of raw data was performed in MATLAB (MathWorks, Natick, Massachusetts, United States) using custom-made scripts, before being transferred to R (R Foundation for Statistical Computing, Vienna, Austria) for statistical analysis. Individual kinematic plots were visually inspected by two raters independently (SM and BN) to identify spurious data points for removal (defined as peak angular kinematics that were considered implausible and resulting from marker occlusion during data collection). A non-parametric approach was taken for the analysis of all data. This was due to multiple violations (i.e., heterogeneity of variance and non-normality of residuals) of the planned parametric approach (i.e., paired *t*-test) across the different variables and time-points. Specifically, 5,000 bootstrap resamples of the paired mean difference (pre-post; within-participant) were used to create a resampling distribution. From this, the mean and 95% confidence interval were calculated and reported. The bias-corrected and accelerated bootstrap was used to account for any skew whilst obtaining the central 95% of the distribution. The “dabestr” package in R was used for statistical analysis and production of figures (33).

## 3. Results

Twenty-one participants were recruited into the study. 18 participants completed the two-week intervention (60% females, mean age 30.6 years ±7.0, height 173.4 cm ±10.4, mass 70.2 kg ±12.4, symptom duration 39.0 months ±58.8) and were able to attend the follow up assessment within the two to three week time window after initial assessment (three participants were unable to attend the follow-up session due to work commitments that prevented attendance at the human performance laboratory). Following a blind data cleaning process to eliminate spurious data values, between 16 and 10 participants data were maintained for analysis of biomechanical variables (see Figure 4 and Supplementary files for participants per analysis).

### 3.1. Symptoms, function and strength

Following the two-week neuromuscular intervention, minimal improvements were observed for VAS, Kujala, and Orebro questionnaire scores. Similarly, minimal change in strength scores were observed (Table 1). Across all clinical measures, the 95% bootstrapped CI of the mean difference contained the null hypothesis (i.e., mean difference = 0).

**Table 1:** Demonstrating the pre-post scores for pain (VAS, Visual Analog Scale), function (Kujala and Tegner scales), psychosocial wellbeing (Orebro), strength (Hip extension and hip External Rotation).

Variable	Pre	Range	Post	Range	Bootstrap estimation		
					Mean difference	−95% CI	+95% CI
VAS	4	*(2–8)*	4	*(1–7)*	−0.7	−1.9	0.4
Kujala	75.5	*(45–95)*	81	*(53–96)*	3.6	−3.8	11.1
Tegner	5	*(3–8)*	5	*(3–8)*	0.0	−0.9	0.8
Orebro	63.5	*(43–101)*	62	*(45–88)*	−6.1	−15.6	2.9
Hip Ext (kg)	13.7	*(7.8–23.3)*	14.9	*(8–27.2)*	2.09	−1.27	5.51
Hip ER (kg)	9.8	*(4.3–13.6)*	9.3	*(5.5–15.3)*	−0.58	−1.34	2.51

### 3.2. Muscle activation onset

No changes in muscle activation onset relative to foot strike were observed during running or stepping down (Table 2). During the step-up task, GlutMed and UGlutMax activated earlier following the two-week neuromuscular intervention, with the GlutMed 95% CI not containing the null hypothesis.

**Table 2:** Mean difference and 95% confidence intervals (CI) of muscle activation onsets (s) relative to foot strike during running, step up and step down tasks.

Variable	Run		Step up		Step down	
	Mean difference	95% CI interval	Mean difference	95% CI interval	Mean difference (s)	95% CI interval
GlutMed	0.00	(−0.02, 0.01)	−0.14	(−0.27, −0.01)	−0.06	(−0.14, 0.01)
LoGlutMax	0.00	(−0.03, 0.03)	0.03	(−0.12, 0.2)	−0.05	(−0.22, 0.04)
TFL	0.00	(−0.02, 0.01)	0.02	(−0.11, 0.16)	−0.03	(−0.10, 0.05)
UpGlutMax	0.00	(−0.02, 0.02)	−0.13	(−0.3. 0.07)	−0.07	(−0.19, 0.02)
VL	−0.01	(−0.02, 0.01)	−0.05	(−0.12, −0.002)	0.01	(−0.08, 0.09)
VMO	0.00	(−0.02, 0.01)	0.00	(−0.10, 0.11)	−0.03	(−0.17, 0.12)

GlutMed, gluteus medius; LoGlutMax, lower gluteus maximus; TFL, tensor fascia lata; UpGlutMax, upper gLuteus maximus; VL, vastus lateralis; VMO, vastus medialis oblique.

### 3.3. Muscle activation

Across all three conditions, and all six muscles investigated, the 95% bootstrapped CI contained the null hypothesis in all instances (Table 3).

**Table 3:** Mean difference and 95% confidence intervals (CI) for peak amplitudes (mV) during running, step up and step down tasks.

Variable	Run		Step up		Step down	
	Mean difference	95% CI interval	Mean difference	95% CI interval	Mean difference (mV)	95% CI interval
GlutMed	20	(−46, 132)	−7	(−37, 23)	−9	(−40, 13)
LoGlutMax	56	(−89, 20)	−8	(−127, 87)	−2	(−46, 36)
TFL	7	(−45, 89)	11	(−67, 85)	7	(−17, 29)
UpGlutMax	−10.50	(−88, 63)	−16	(−48, 13)	−7	(−26, 11)
VL	−1	(−155, 134)	−2	(−92, 88)	−10	(−125, 52)
VMO	16	(−126, 175)	−17	(−138, 116)	2	(−146, 112)

GlutMed, gluteus medius; LoGlutMax, lower gluteus maximus; TFL, tensor fascia lata; UpGlutMax, upper gLuteus maximus; VL, vastus lateralis; VMO, vastus medialis oblique.

### 3.4. Kinematic measures

During running, no changes in knee flexion, hip adduction, or knee rotation were observed following the two-week neuromuscular intervention (Table 4). Small increases in knee flexion during step-up and step-down were observed, but the 95% bootstrapped CI still contained the null hypothesis. No differences in hip adduction or knee rotation were observed during all tasks.

**Table 4:** Mean difference and 95% confidence intervals (CI) for peak joint angles during running, step up and step down tasks.

Variable	Run		Step up		Step down	
	Mean difference	95% CI interval	Mean difference	95% CI interval	Mean difference (°)	95% CI interval
KneeRot	0.28	*(−8.0, 8.6)*	−1.74	*(−11.5, 6.8)*	−0.11	*(−12.7, 10.5)*
KneeFlex	−0.27	*(−4.7, 3.9)*	2.73	*(−0.9, 6.2)*	9.02	*(−0.03, 22.9)*
HipAdd	1.01	*(−2.0, 4.0)*	1.27	*(−2.4, 3.7)*	1.49	*(−1.4, 5.0)*

KneeRot, knee rotation; KneeFlex, knee flexion; HipAdd, hip adduction.

## 4. Discussion

The aim of this study was to evaluate the effects of a two-week neuromuscular intervention, directed at the hip muscles, on biomechanical and psychosocial variables in people with PFP. After this short intervention, only minimal changes in measures of biomechanical or psychosocial variables were identified, but indications for directions of change were evident. The absence of any large changes may relate to the relatively short duration of the intervention and the high variability that was evident in the biomechanical variables, particularly measures of muscle electromyography.

The Orebro questionnaire was adopted to better understand the psychosocial characteristics of symptom persistence in this group, relevant given long average symptom duration reported in the literature (34) and evident within this cohort (39 ± 58.8 months). The psychosocial features evaluated through the use of the Orebro questionnaire did not change following the two-week intervention. The psychometric properties of the Orebro questionnaire align with determinants of persistent pain. Whilst these determinants may be anxiety and fear avoidance, there are a much wider set of characteristics that are being measured with this tool. In only one participant was the pre-defined questionnaire threshold (>105)—for predicting recovery in individuals with low back pain—exceeded (26). The absence of change in Orebro score may be related to the lack of sensitivity for the Orebro questionnaire to specifically identify elevated levels of anxiety and fear avoidance reported to be evident in this patient population (35), or the limited severity of problems evident within this cohort.

Kinematics of the hip and knee have been extensively evaluated in people with PFP, with variance from pain free individuals observed in sagittal and frontal plane movements during activities such as running and stair ascent or decent (13). The determinants of these adopted movement patterns have been postulated to result from pain experienced during the activity or deficits in their muscular control (36). The ability of rehabilitative exercise, targeting the hip and knee muscles, to affect change in muscular and subsequent kinematic control remains plausible, but the intervention delivered in this study was insufficient to achieve this outcome. Whilst neuromuscular modulation can be achieved during a short intervention period, it did not result in a change in symptoms and this may explain why kinematic change was not observed. A longer intervention period that incorporates progressive overload principles of training is likely to be necessary and represents a research priority to ensure that understand the mechanism through which readily prescribed exercise interventions are having their effect.

Delayed onset and shorter activation duration of gluteal musculature has been observed in people with PFP (10). Strength deficits of both the hip abductors and extensors in people with pain are also consistently reported and as such represent clear treatment targets for the management of this population (8). The absence of change following the two-week intervention in both gluteal onset time and RMS observed in this study may be attributable to the inherent challenges associated with onset determination (37), but also the duration and intensity of the delivered intervention. Very limited evidence exists for the magnitude of load required to achieve changes in biomotor properties of the working muscle, with only high intensity training loads having been shown to affect change within these muscle characteristics (38). Further work is needed to investigate whether rehabilitation interventions of longer durations or working at higher load magnitudes for short durations are sufficient to achieve biomotor change in a symptomatic population.

Traditional training paradigms purport that changes in muscle force output in the short term may be attributable to changes in neural excitation of the activated muscle (39). Bolgla et al. reported that responders to a six-week rehabilitative exercise programme achieved strength changes of 5% (females) and 15.4% (males) body mass for hip external rotation and 13.1% (females) and 14.6% (males) body mass for hip extension (40). Our participants achieved increases in hip extension strength, but a mean reduction in hip external rotation strength following the two-week intervention. These small and inconsistent changes in hip strength may provide further evidence for why no changes were observed in the biopsychosocial variables measured in this study. Although increases in hip strength has been shown to not mediate patient reported outcomes following an exercise intervention (41), changes in strength have not been explored as a mediator for change in psychosocial outcomes. Investigations to better understand through what mechanism exercise interventions are having their positive effects in people with PFP are needed.

A limitation of the study was the lower sample size for the biomechanical data following the removal of data that was visually determined to be spurious. Despite the a-priori sample having been recruited, the lower sample means caution is necessary when interpreting the biomechanical outcomes. The statistical methods adopted sought to mitigate this risk, through applying a robust bootstrapping method (33, 42). The overall sample size should be considered with respect to the potential for type II error (i.e., false negatives) when interpreting our results. The potential for EMG crosstalk should also be acknowledged, but we mitigated this risk by placing our electrodes using the SENIAM guidelines. We also acknowledge that we did not perform an independent reliability analysis of our EMG methods as we did for our kinematic methods. The chosen outcome measure represents a further limitation of the study. Whilst the psychometric properties of the Orebro questionnaire align well with prediction of symptom chronicity, they do not specifically seek to understand the specific psychosocial variables that have previously been reported to be associated with PFP. Pain levels during the functional tasks were not captured, limiting the ability to make conclusions regarding the impact of pain on the biomechanical measures taken. Subsequent studies should aim to ensure that pain levels are captured during functional tasks and that the outcome measurement tool accurately evaluates anxiety, pain catastrophising and fear avoidance behaviours to maximise the external validity of the results. Participants received no further contact with a physiotherapist between baseline and follow up, which may have negatively affected their adherence, but we mitigated this with our use of exercise sheets and diaries.

## 5. Conclusions

A two-week neuromuscular intervention, directed at the hip musculature of people with PFP, did not result in significant change to measures of biopsychosocial characteristics. The changes that were evident may indicate that interventions of longer durations or greater load magnitudes could be sufficient to elicit change. Further studies to investigate mechanisms of effect for exercise interventions should continue to evaluate psychosocial, as well as biomechanical, variables to best inform the future management of this recalcitrant condition.

## Data Availability

The raw data supporting the conclusions of this article will be made available by the authors, without undue reservation.
