# Supplementary figures and images for: The effects of a two-week neuromuscular intervention on biopsychosocial variables in people with patellofemoral pain: an observational study
Source: Front Sports Act Living. 2023 May 15;5:1087061. doi: 10.3389/fspor.2023.1087061 (PMC10225691; doi:10.3389/fspor.2023.1087061)

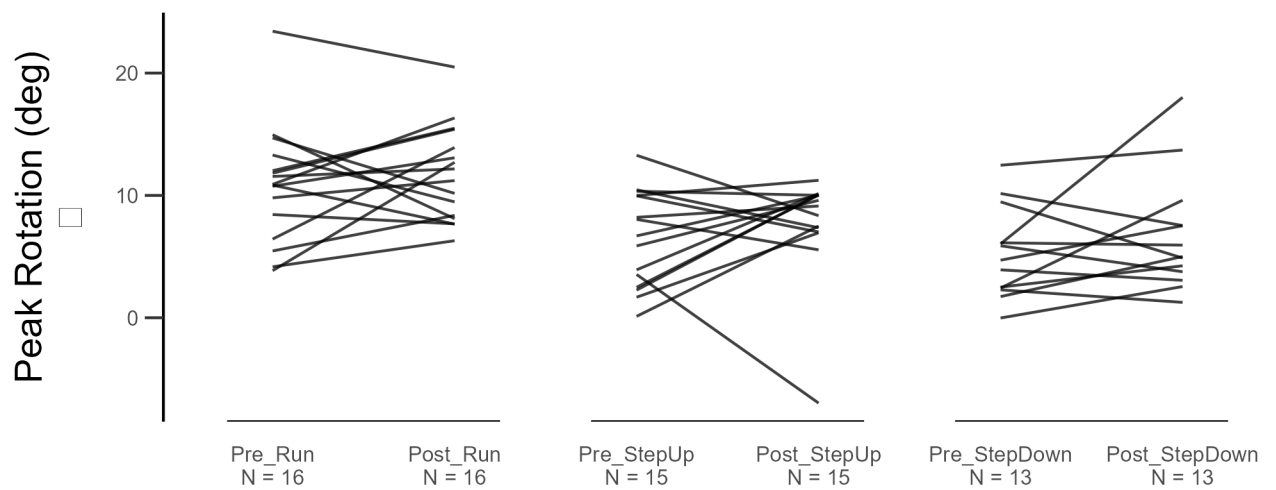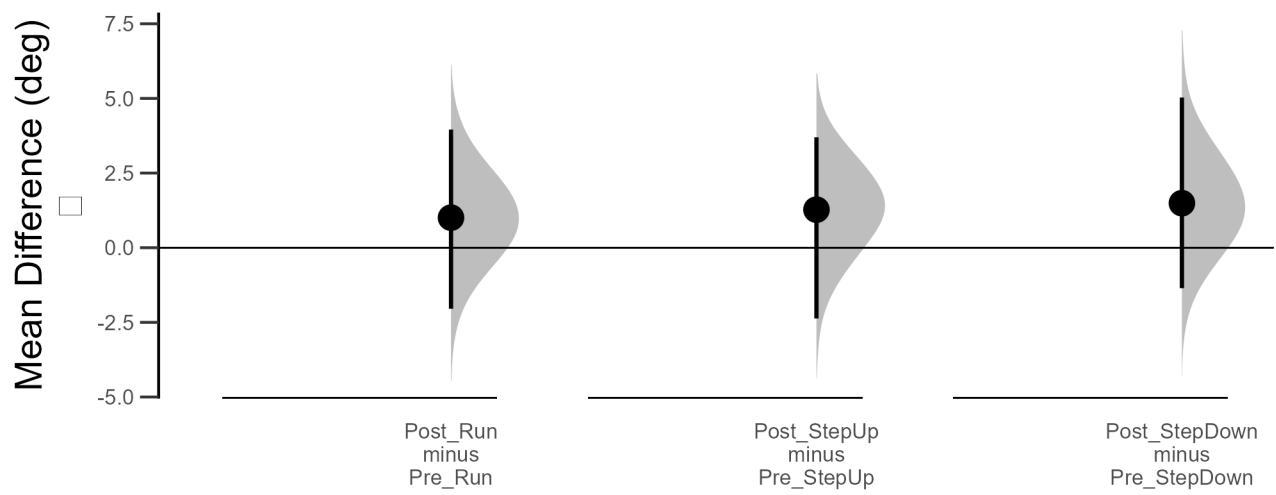

Supplement: Supplementary file 1 [file Image1.pdf]

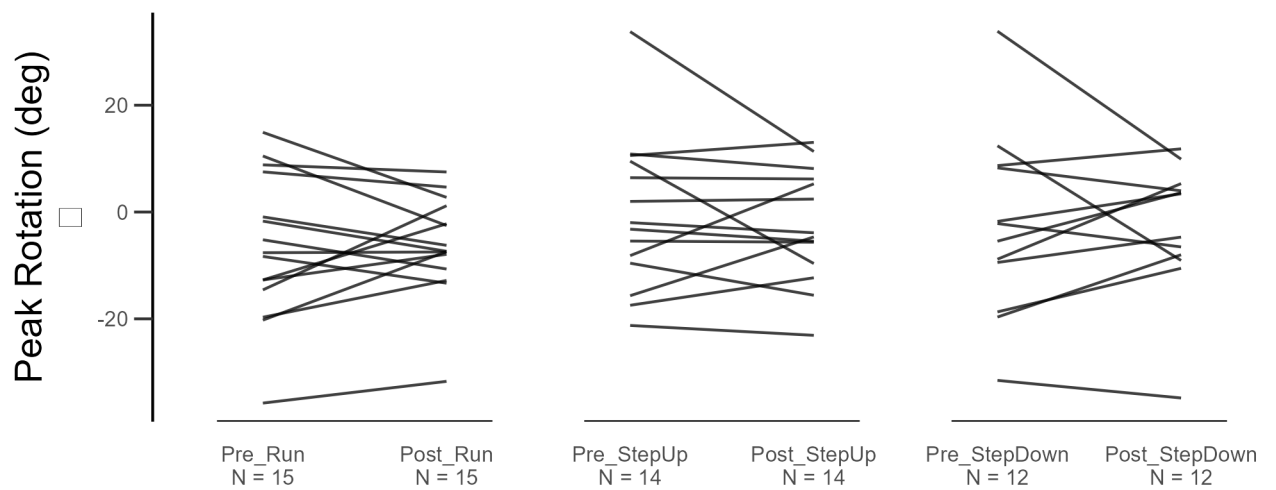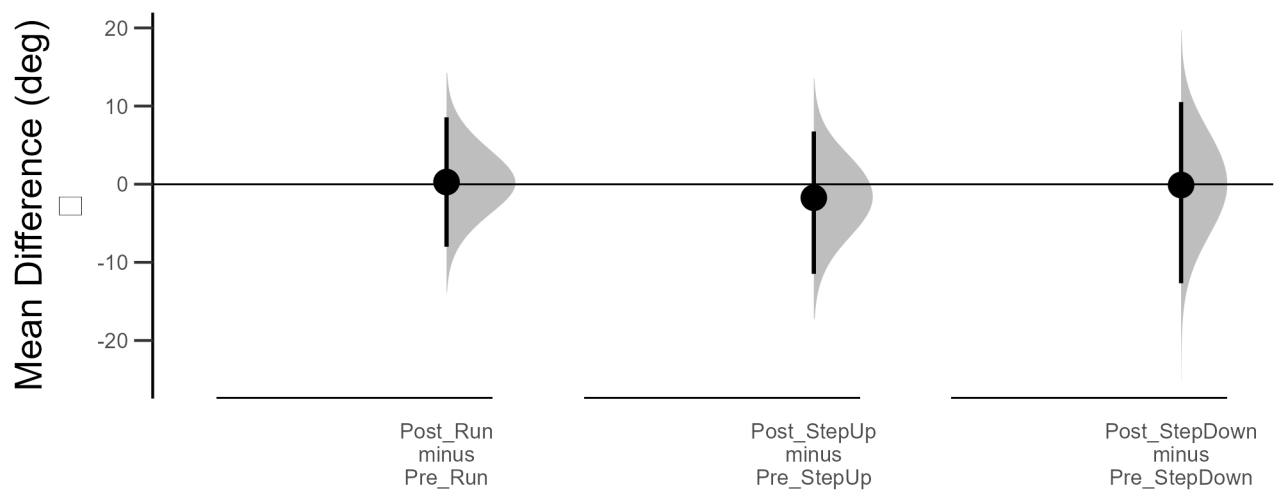

Supplement: Supplementary file 2 [file Image2.pdf]

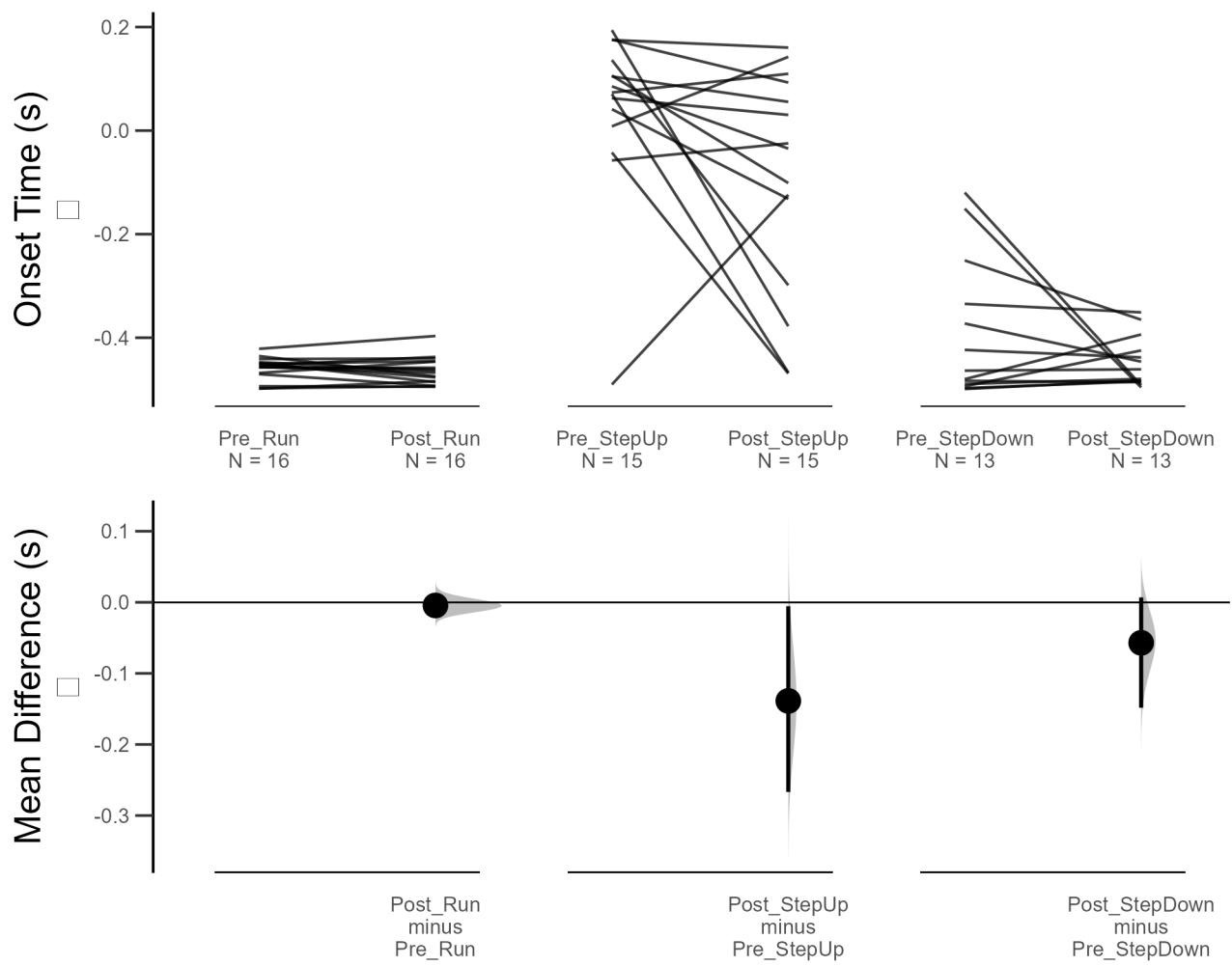

Supplement: Supplementary file 3 [file Image3.pdf]

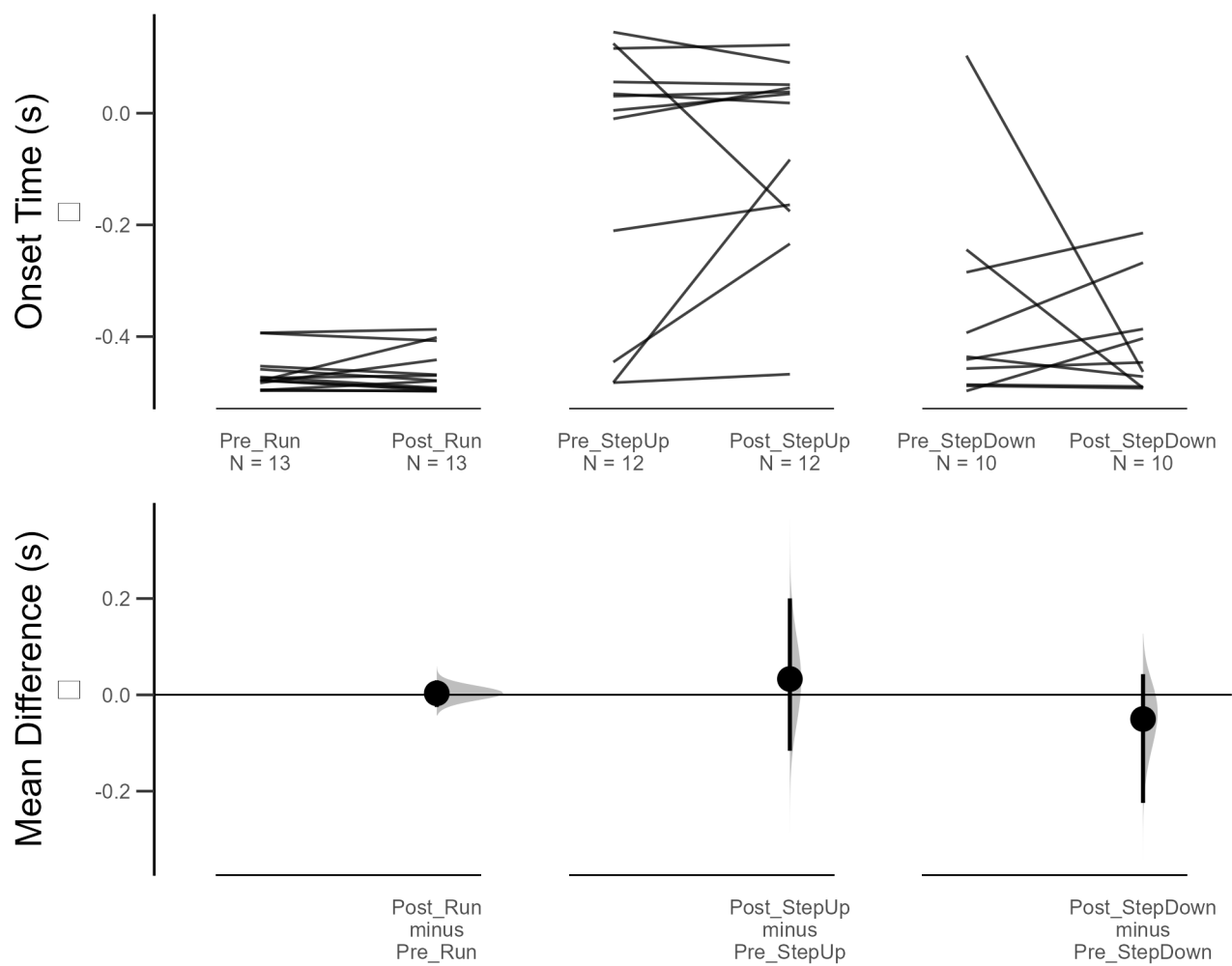

Supplement: Supplementary file 4 [file Image4.pdf]

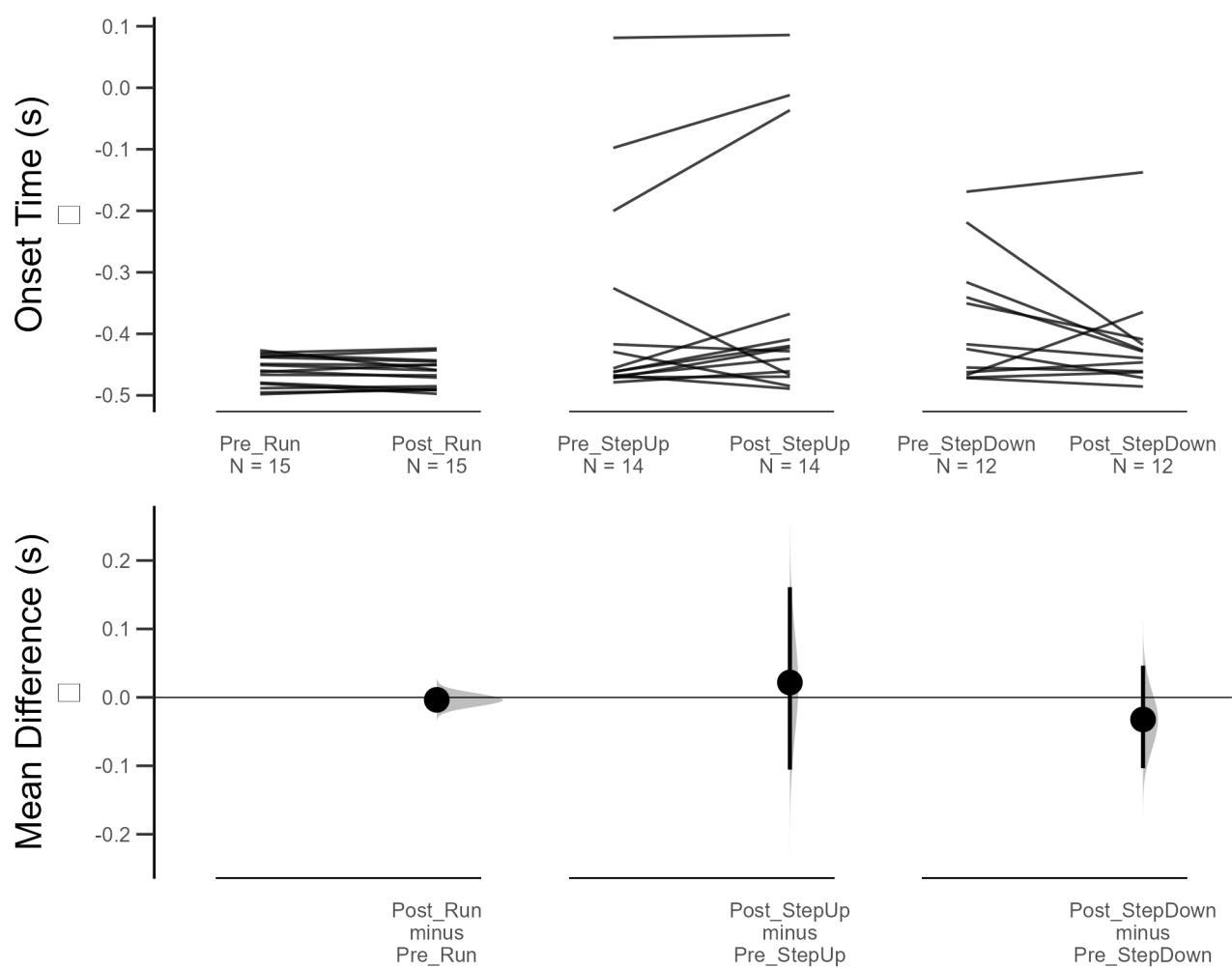

Supplement: Supplementary file 5 [file Image5.pdf]

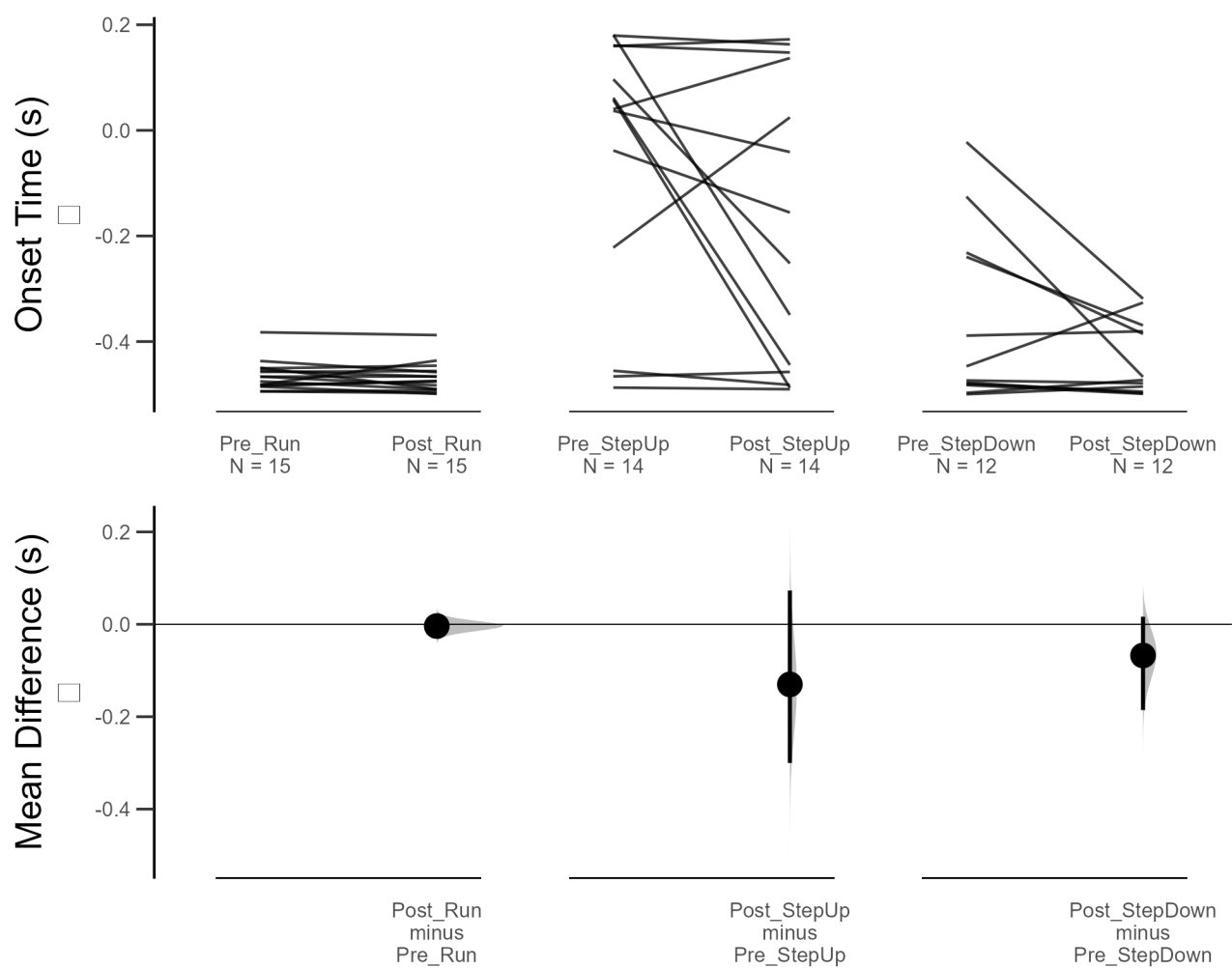

Supplement: Supplementary file 6 [file Image6.pdf]

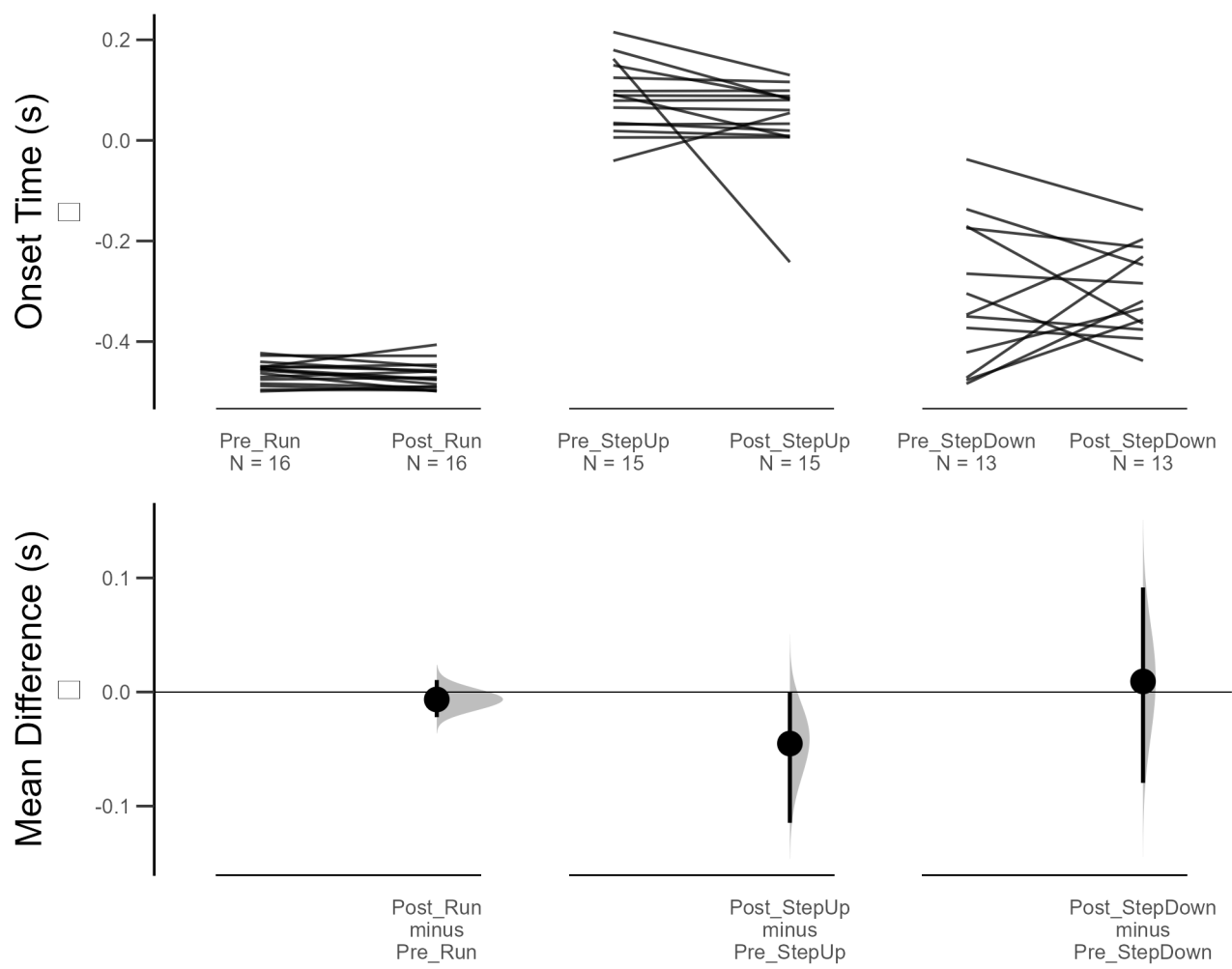

Supplement: Supplementary file 7 [file Image7.pdf]

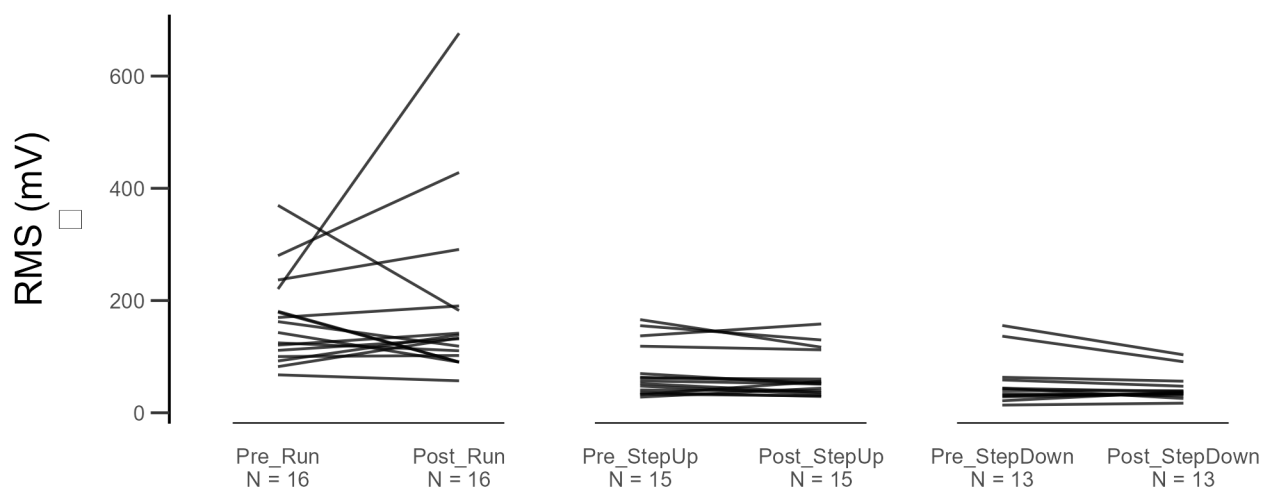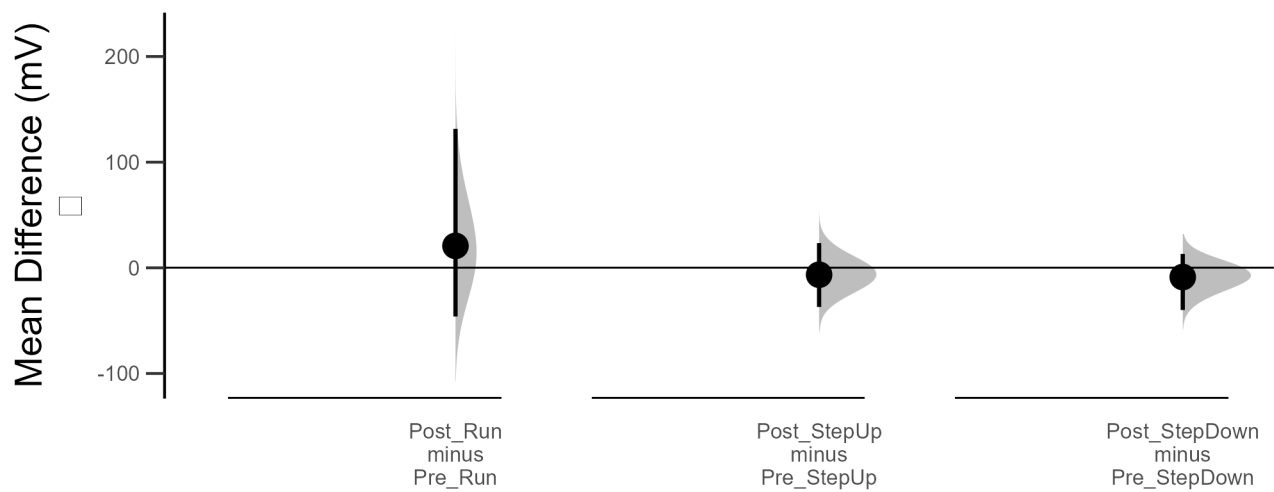

Supplement: Supplementary file 8 [file Image8.pdf]

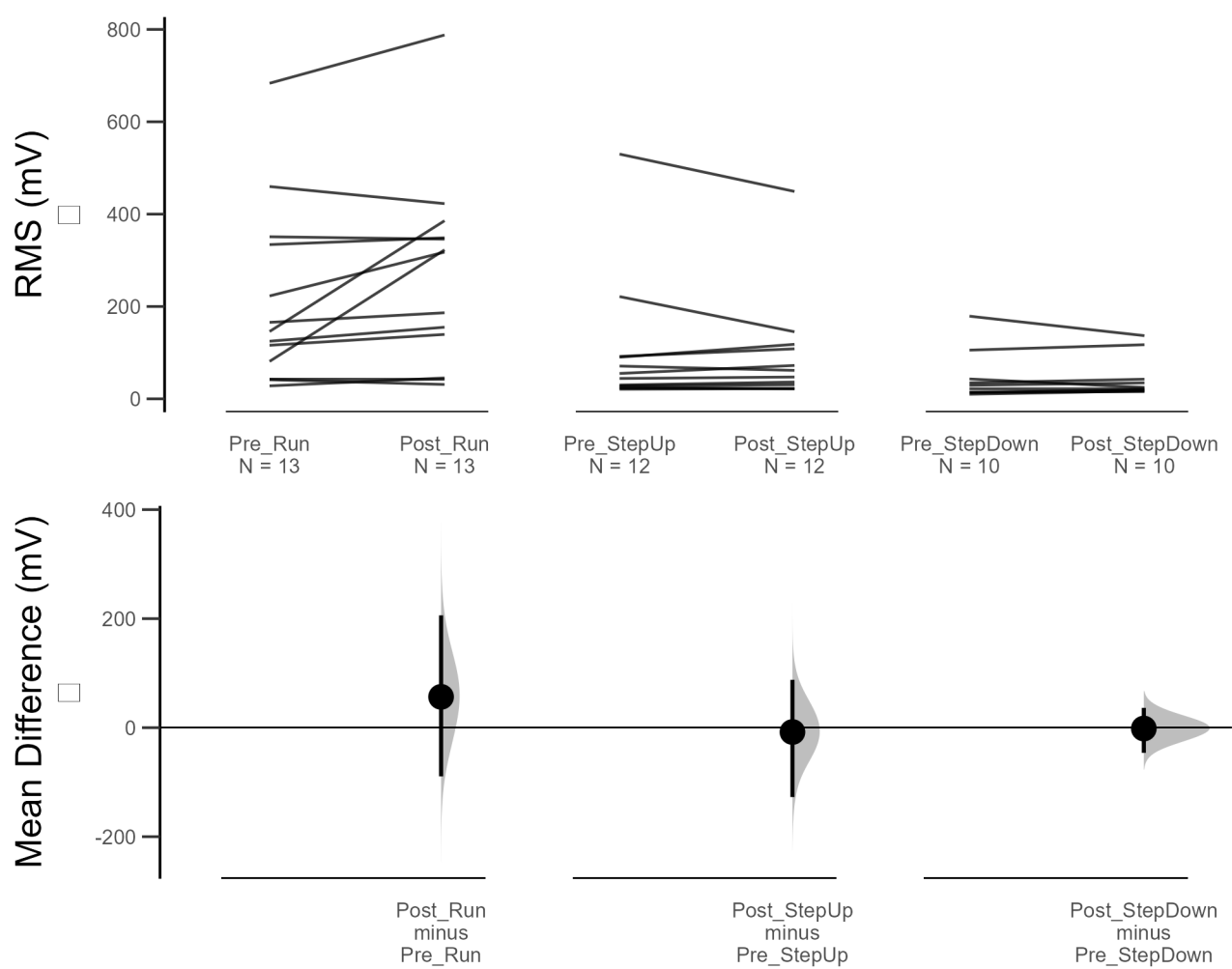

Supplement: Supplementary file 9 [file Image9.pdf]

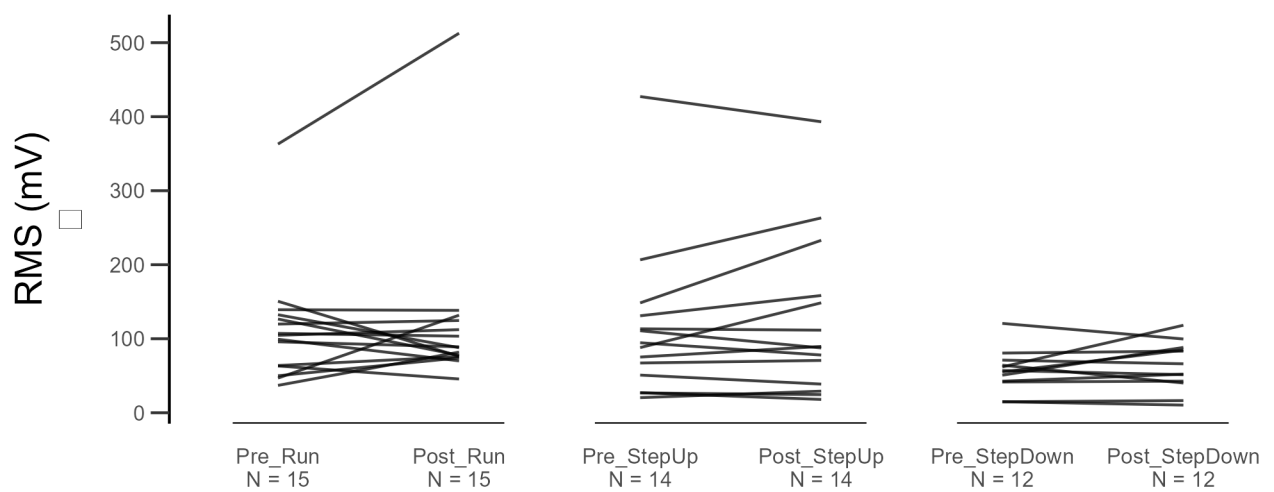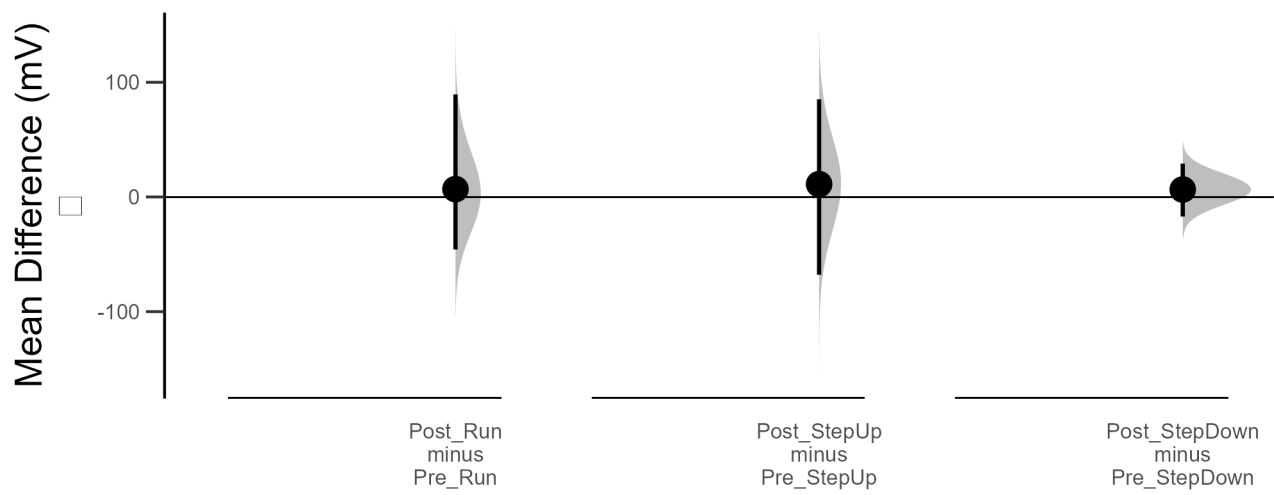

Supplement: Supplementary file 10 [file Image10.pdf]

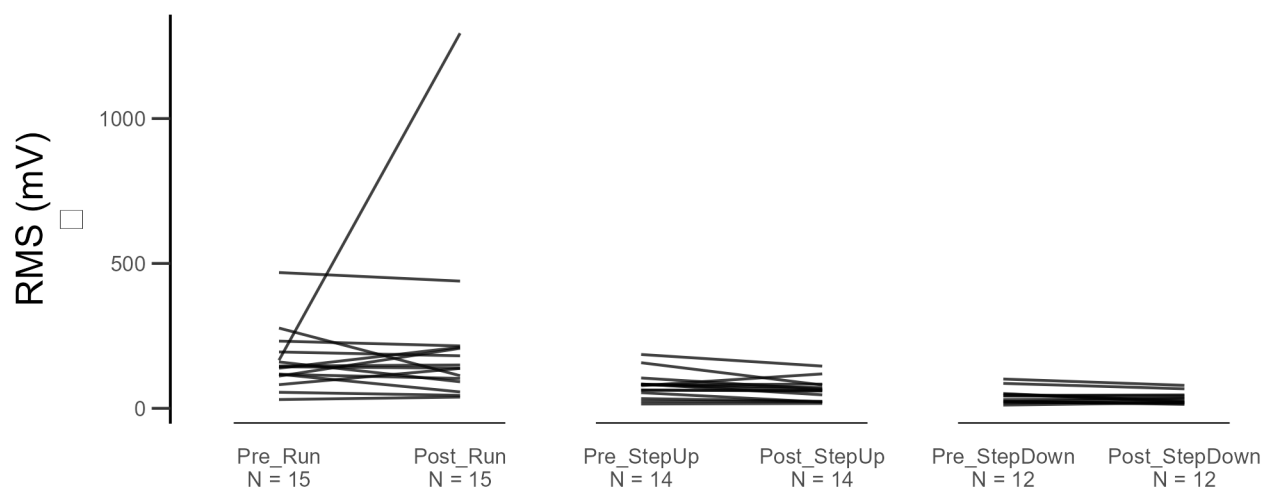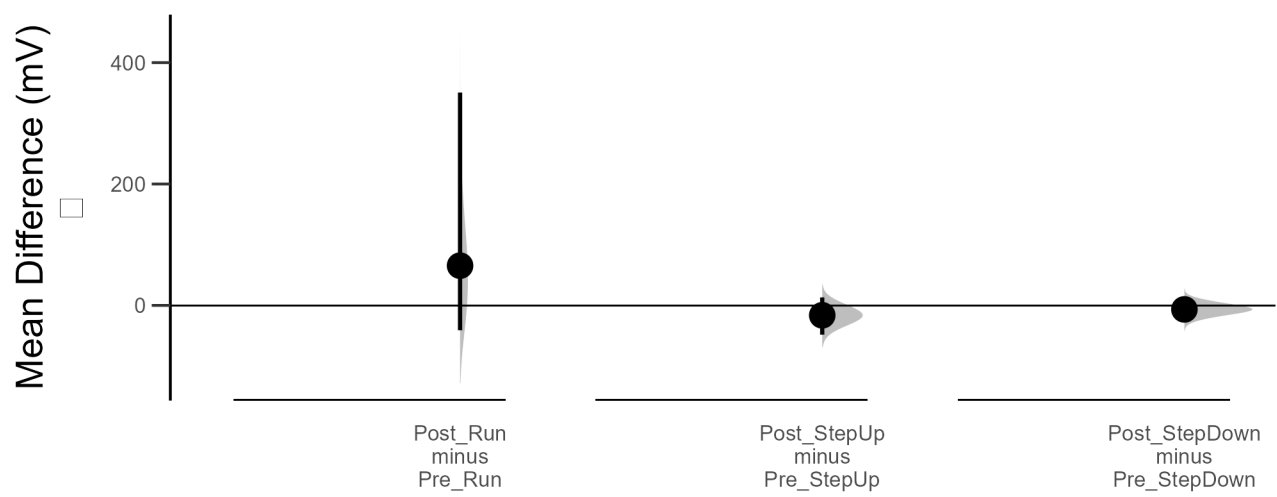

Supplement: Supplementary file 11 [file Image11.pdf]

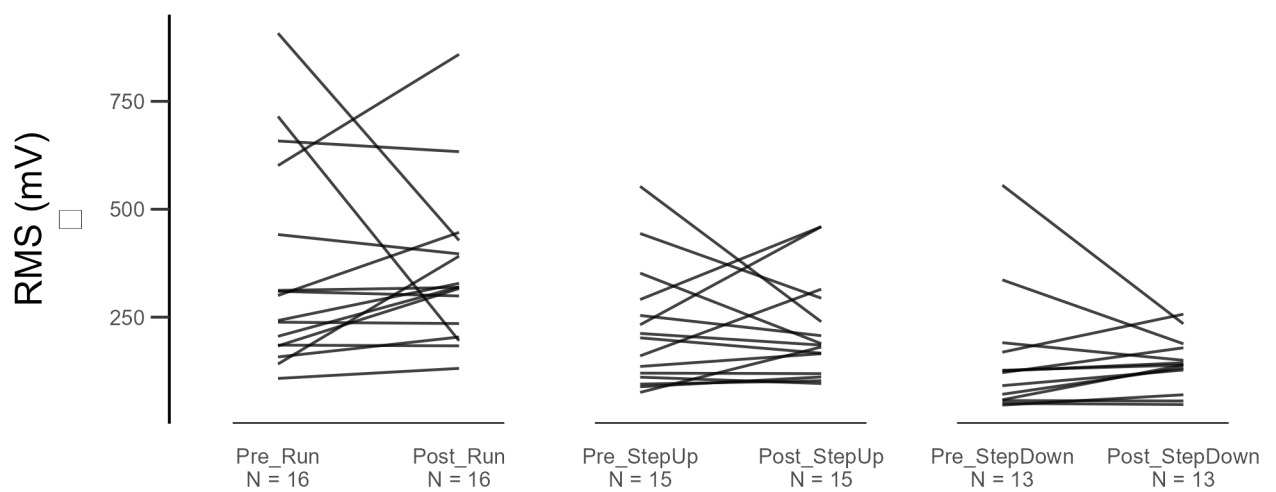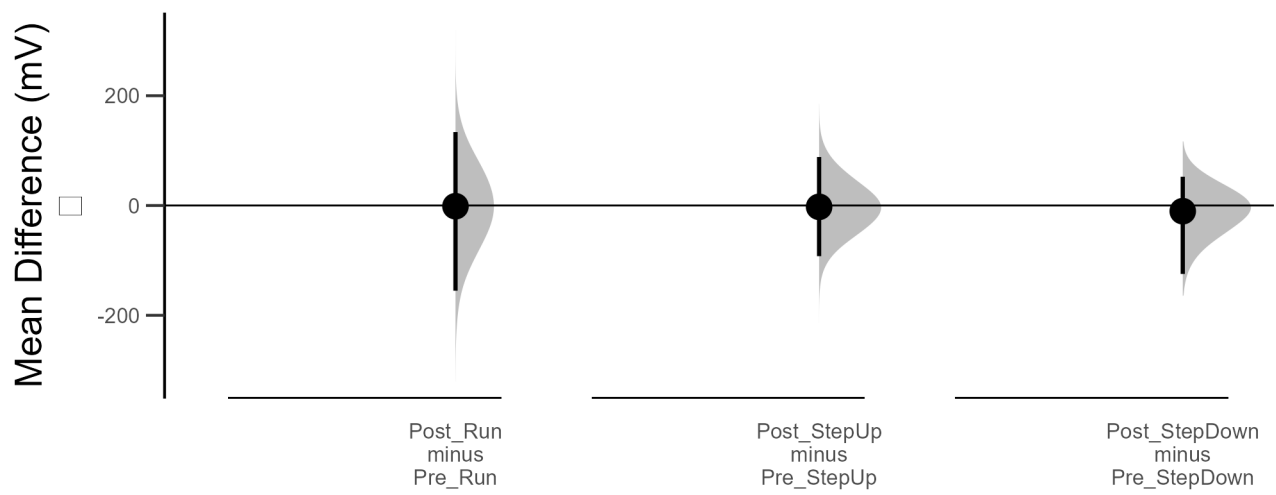

Supplement: Supplementary file 12 [file Image12.pdf]

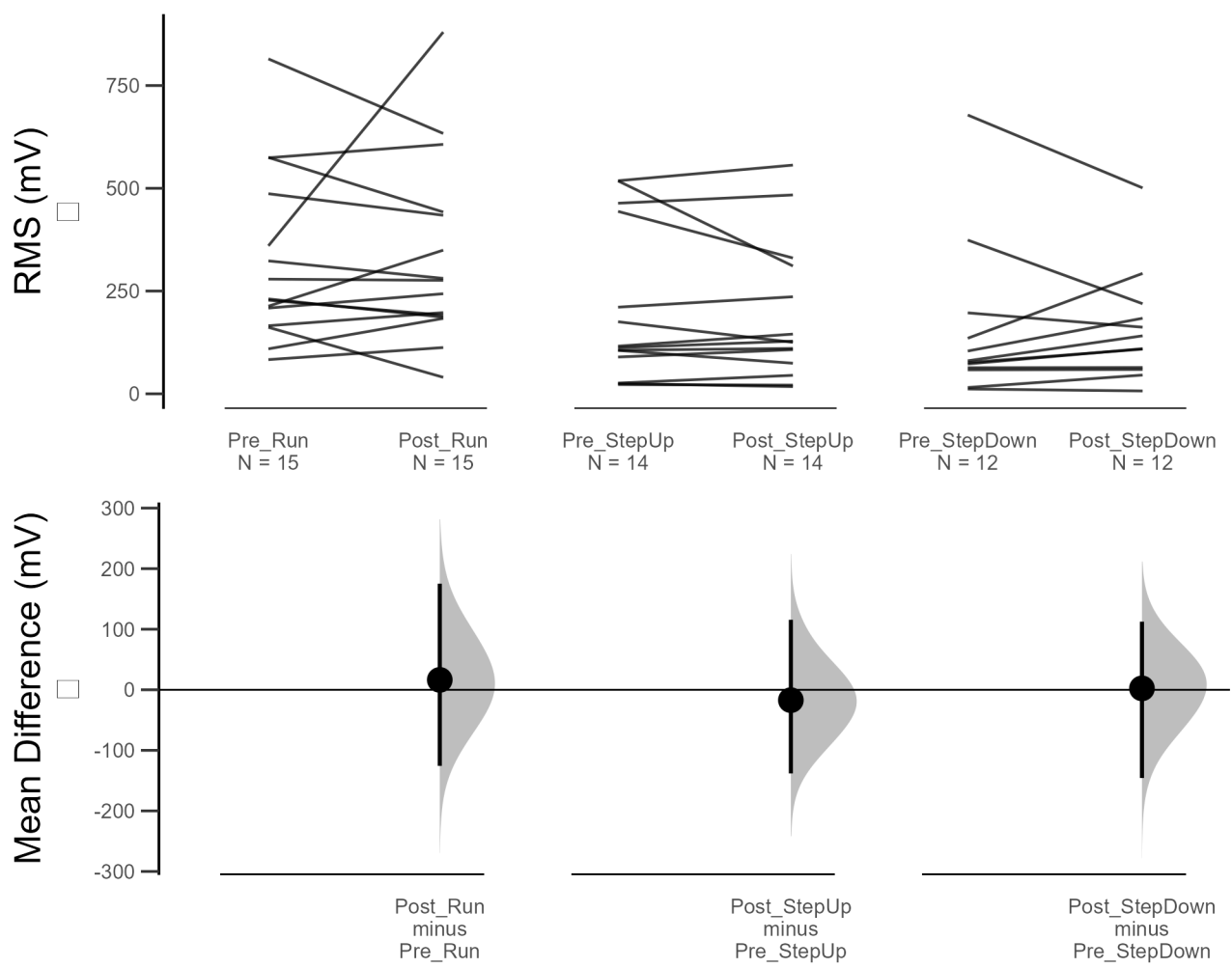

Supplement: Supplementary file 13 [file Image13.pdf]
